# Metastasis-associated gene 1 promotes invasion and migration potential of laryngeal squamous cell carcinoma cells

**DOI:** 10.3892/ol.2013.1729

**Published:** 2013-12-05

**Authors:** HAILI ZHANG, DONG YANG, HAIJUAN WANG, SHUXIN WEN, JIAN LIU, QINGCHUN LUAN, YIXUAN HUANG, BINQUAN WANG, CHEN LIN, HAILI QIAN

**Affiliations:** 1 Department of Otolaryngology Head and Neck Surgery, The First Hospital, Shanxi Medical University, Taiyuan, Shanxi 030001, P.R. China; 2 State Key Laboratory of Molecular Oncology, Cancer Institute/Cancer Hospital, Chinese Academy of Medical Sciences and Peking Union Medical College, Chao Yang, Beijing 100021, P.R. China

**Keywords:** metastasis-associated gene 1, laryngeal squamous cell carcinoma, invasion, metastasis

## Abstract

Overexpression of the metastasis-associated gene 1 (*MTA1*) has previously been found to be associated with progression of various cancer types to the metastasis stage. The function of *MTA1* in laryngeal squamous cell carcinoma (LSCC) remains unclear. To explore the significance of *MTA1* in the invasion and migration processes in LSCC, gene transfection and RNA interference (RNAi) were performed to study the biological function of *MTA1* in the LSCC cell line, HEP-2. Results showed that *MTA1* promoted the invasion, adhesion and migration behavior of LSCC cells. RNAi against *MTA1* significantly decreased the malignant phenotypes of cancer cells. *MTA1* may be important in the process of LSCC invasion and metastasis.

## Introduction

Laryngeal squamous cell carcinoma (LSCC) represents the second most common malignant neoplasm of the respiratory tract after lung cancer (1). LSCC has a strong propensity to metastasize to regional lymph nodes, which decreases the cure and survival rates (2,3). Multiple steps and factors are involved in the process of malignant cancer progression (4,5). This process is under the control of many metastasis-associated genes. Among these, metastasis-associated gene 1 (*MTA1*) is positively correlated with cancer metastasis in many cancer types. Toh *et al*(6) first cloned *MTA1* from the highly metastatic mammary adenocarcinoma cell line by differential cDNA library screening. *MTA1* is a subunit of the nucleosome remodeling and deacetylase (NURD) complex, which is involved in chromatin remodeling and histone deacetylation in gene expression regulation (7). *MTA1* functions as a transcriptional coregulator, regulating the downstream target genes that encode effector proteins controlling cancerous processes (8). *MTA1* overexpression is positively correlated with *in vitro* migration and invasion ability in KYSE150 and B16F10 melanoma cell lines, and inhibition of *MTA1* protein expression results in growth inhibition of cancer cell lines (9,10). Sasaki *et al*(11,12) reported that *MTA1* mRNA was overexpressed in thymoma and advanced lung cancer. It has also been reported that *MTA1* may be involved in initiating carcinogenesis (13,14). Miyatani *et al*(15) compared the expression of *MTA1* in normal esophageal epithelium, normal gastric epithelium and gastro-esophageal junction cancer, and found that *MTA1* levels were significantly higher in cancer samples than in their normal counterparts. Moreover, in tonsil cancer, *MTA1* is positively correlated with lymphatic metastasis (16). It has also been indicated that *MTA1* is correlated with tumor angiogenesis and poor outcome in patients with early-stage non-small cell lung cancer (NSCLC) (17). This line of evidence indicates that *MTA1* may become a new marker for predicting cancer metastasis, or even cancer outcome.

Concerning the molecular mechanism of *MTA1* in cancer cell metastasis, *MTA1* has been reported to be involved in cancer development in several ways. *MTA1*-interacting coactivator has been identified as a molecule that interacts with *MTA1* to regulate estrogen receptor-α transactivation (18). Yoo *et al*(19) reported that *MTA1* stabilizes hypoxia-inducible factor-1α protein by recruiting histone deacetylase 1, and is correlated with angiogenesis in cancer development (20). Since *MTA1* is a histone deacetylase (HDAC)-interacting protein that modulates the epigenetic status of its target genes, it is expected to widely influence the expression pattern of the cancer-related gene spectrum. Ghanta *et al*(21) revealed, using a profiling assay, that *MTA1* regulation was partially under the control of p53. When p53 is functional, *MTA1* mainly focuses on inflammatory and antimicrobial responses; when p53 is absent, *MTA1* predominantly targets genes in cancer signaling. *MTA1* is correlated with cigarette smoking in NSCLC, indicating its importance in the smoking-related progression of this type of cancer (22). *MTA1* has also been reported to regulate the anoikis of human prostate cancer cells (23), which reveals a new subfield of *MTA1* mechanisms.

*MTA1* is a corepressor responsible for estrogen receptor repression at the transcriptional level (24). A naturally occurring *MTA1* variant, *MTA1*s, can sequester estrogen receptor-α in the cytoplasm (25). Estrogen receptor involvement is the first insight into the p53-independent function of *MTA1* in the DNA damage response involving the p21/WAF1-proliferating cell nuclear antigen pathway (26). *MTA1* is required for the ATR-mediated DNA damage checkpoint function (27). UV radiation stabilizes *MTA1* and increases *MTA1* binding to ATR. Other molecules found to be associated with *MTA1* expression include RECK (28), HDAC1 (15) and MMP-9 (29). Silencing *MTA1* by RNA interference (RNAi) reverses the malignant phenotypes, including adhesion, migration and invasiveness of cervical cancer cells (SiHa) via altered expression of p53 and the E-cadherin/β-catenin complex (30).

No systematic biological studies have been performed on LSCC to date. This study aimed to determine the biological role of *MTA1* in LSCC using gain-of-function and RNAi techniques.

## Materials and methods

### Cell lines

The human LSCC cell line HEP-2 and the human keratinocyte HaCaT cell line (State Key Laboratory of Molecular Oncology, Beijing, China) were cultured in RPMI-1640 and DMEM medium (Gibco-BRL, Grand Island, NY, USA), respectively, supplemented with 10% (v/v) fetal calf serum (FCS) (Hyclone Laboratories, Inc., Logan, UT, USA), 2 mM L-glutamine and antibiotics (penicillin-streptomycin at 100 U/ml) in a humidified atmosphere of 5% CO_2_ at 37°C. The keratinocyte HaCaT cell line is an immortalized normal epithelial cell line.

### Reagents

Lipofectamine 2000 was purchased from Invitrogen Life Technologies (Carlsbad, CA, USA). siRNA sequence was chemically synthesized by Jikai Co. (Shanghai, China). The MTA1 primary antibody was from Santa Cruz Biotechnology, Inc. (sc-9446; Santa Cruz, CA, USA) and the horseradish peroxidase-conjugated secondary antibody was from Zhongshan Biotech, Co., Ltd (Zhongshan, China). The ECL detection system was purchased from Amersham Biosciences (Piscataway, NJ, USA). The Boyden chamber system and polycarbonate membrane (8 μm pore size) were obtained from Neuro Probe, Inc. (Canada). Matrigel was purchased from BD Biosciences (San José, CA, USA).

### siRNA and plasmid transfection

The 21-nt siRNA sequence was chemically synthesized (Jikai Co.). The target sequences of the siRNA for the *MTA1* gene (*MTA1*-siRNA) were as follows: Sense, 5′-GAACAUCUACGACAUCUCCdTdT-3′ and antisense, 5′-GGAGAUGUCGUAGAUGUUCdTdT-3′ (9). The *MTA1*-siRNA was dissolved in sterilized and RNase-free water and annealed. The final concentration was 20 μM. Lipofectamine 2000 (20 μl/ml, Invitrogen Life Technologies) was used to transfect the HEP-2 cell line according to the manufacturer’s instructions. A sequence non-specific to any known gene was used as a negative control (Jikai Co.). The pcDNA3-*MTA1* plasmid was provided by Dr Mahoney (Jefferson Institute of Molecular Medicine, Thomas Jefferson University, Philadelphia, PA, USA), and the transfection was performed according to the manufacturer’s instructions. The cells transfected with pcDNA3-*MTA1* were selected by G418 prior to use.

### Western blotting analysis

Cells were grown to 80% confluence and rinsed twice with 1X PBS prior to harvesting. Total cell protein was extracted using PBS buffer containing aprotinin (2 μg/ml), PMSF (100 μg/ml), leupeptin (2 μg/ml) and 1% Nonidet P-40. Protein concentration was determined using the Gene Quant Pro-91738 protein assay system (Bio-Rad Laboratories, Inc, Hercules, CA, USA). Samples were briefly electrophoresed in 10% SDS-PAGE and transferred to nitrocellulose membranes using a semidry transfer system. Nonspecific binding was blocked for 2 h in 5% fat-free milk in PBS buffer, pH 7.6. Blots were first incubated with *MTA1* primary antibody (1:200) (Santa Cruz Biotechnology, Inc.) for 2 h at 37°C, and then with corresponding horseradish peroxidase-conjugated secondary antibody (1:2,000, Zhongshan Biotech) for 1 h at room temperature. Signals were visualized using the ECL detection system according to the manufacturer’s instructions (Amersham Biosciences). The detection was repeated three times.

### Migration and invasion assay

Migration assays were performed using a Boyden chamber system (Neuro Probe, Inc., Gaithersburg, MD USA) with a fibronectin-precoated (0.5 mg/ml) polycarbonate membrane (8 μm pore size) (AP48; Neuro Probe, Inc.) as described previously, with minor modifications (9). The invasion chamber was identical to the migration chamber, but with the 250 μg/ml Matrigel (BD Biosciences) precoated polycarbonate membrane. For both assays, the bottom chambers were filled with medium containing 10% FCS, RPMI-1640 and 2% BSA as chemoattractant, and medium containing serum-free RPMI-1640, and 0.2% BSA was added into the top chambers. Treated or control cells (2×10^4^ per well) were added to the top chambers, followed by a 10-h incubation at 37°C and 5% CO_2_. Three independent experiments were performed for each set. The cells migrated through and adhered to the bottom of the membrane were then fixed and stained with Giemsa dye. The cells that migrated to the lower side of the membrane were mounted under a microscope and averaged. This experiment was repeated three times.

### Adhesion assay

Adhesion assay was performed by the MTT assay. The same numbers of *MTA1*-siRNA-, control-siRNA- and pcDNA3-*MTA1-*transfected HEP-2 cells (1×10^5^) were plated into the Matrigel-precoated (50 μg/ml) 96-well plate in triplicate. The groups of cells were washed for 30, 60 and 90 min, respectively, to remove the non-adherent cells. After washing, the adherent cells were measured with MTT assay at 490 nm wavelength. The OD values reflect the proportion of cells that adhered to the Matrigel-coated 96-well plate. This experiment was repeated in triplicate.

### Wound healing assay

To perform the wound healing assay, pcDNA3-*MTA1-*, *MTA1*-siRNA- and control-siRNA-transfected HEP-2 cells were implanted into the Matrigel (50 μg/ml)-coated 35-mm culture dishes, as described by Qian *et al*(9). When the cells grew to 80% confluence, a sterilized tip was used to draw a line with the same width on the bottom of the dishes. Images were captured at 8, 16 and 24 h after the wounding. Data shown in the text are representative of three independent repeats.

### RT-PCR analysis

Total RNA was isolated from HEP-2 and HaCaT cells, and RT-PCR was performed according to the manufacturer’s instructions to detect gene expression (K0011 RT-PCR kit, Vigorous Biotechnology, Beijing, China). The primers used for amplification were as follows: *MTA1* (forward primer: 5′-CCGGGCCTGCGAGAGCTGTTACAC-3′, reverse primer: 5′-CACGGCTTCCAGCGGCTTGCGTAC-3′); β-actin (forward primer: 5′-ACCACAGTCCATGCCATCAC-3′, reverse primer: 5′-TCCACCACCCTGTTGCTGTA-3′). The cycling conditions were as follows: Initial denaturation at 94°C for 5 min, followed by 28 cycles at 94°C for 30 sec, 55°C for 40 sec and 72°C for 30 sec.

### Statistical analyses

The data were analyzed by ANOVA. The statistical analysis was performed using SPSS 11.0 software (SPSS, Inc, Chicago, IL, USA), and P<0.05 was considered to indicate a statistically significant difference.

## Results

### MTA1 mRNA expression in HEP-2 and HaCaT cell lines

**Figure 1:**
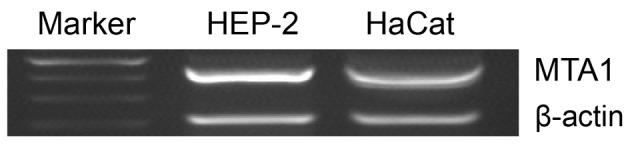
*MTA1* expression in HEP-2 and HaCaT cell lines. Total RNA was extracted from the cultured cell lines and reversely transcribed to cDNA as a template for RT-PCR amplification. The level of *MTA1* mRNA in the HEP-2 cell line was significantly higher than that in the HaCaT cell line. *MTA1*, metastasis-associated gene 1.

Expression of *MTA1* mRNA in Hep-2 and HaCaT cell lines was evaluated by RT-PCR. The level of *MTA1* mRNA was significantly higher in the LSCC cell line, HEP-2, than in the immortalized keratinocyte HaCaT cell line (Fig. 1). This result indicates that LSCC cells have higher levels of *MTA1* expression than normal cell lines, as indicated by the active role of *MTA1* in LSCC. Thus, for further experiments in this study, the HEP-2 cell line was selected to study *MTA1* biological functions.

### Confirmation of MTA1 expression by RT-PCR and western blot analysis in the gain-of-function and loss-of-function studies

**Figure 2:**
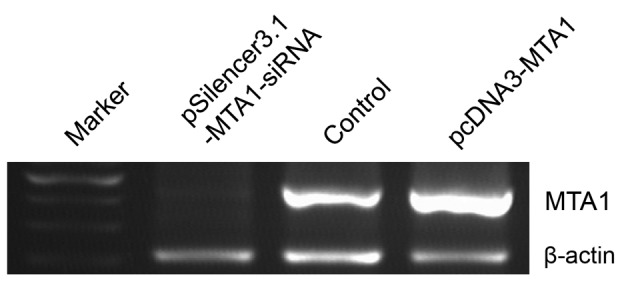
RT-PCR determined the effects of pcDNA3-*MTA1*, control-siRNA and *MTA1*-siRNA transfection on *MTA1* expression at the mRNA level. pcDNA3-*MTA1* and *MTA1*-siRNA were transfected into the cell line with Lipofectamine 2000. Total RNA was extracted and RT-PCR was performed. pcDNA3-*MTA1* transfection increased *MTA1* mRNA levels, while *MTA1*-siRNA decreased *MTA1* mRNA levels. *MTA1*, metastasis-associated gene 1.

**Figure 3:**
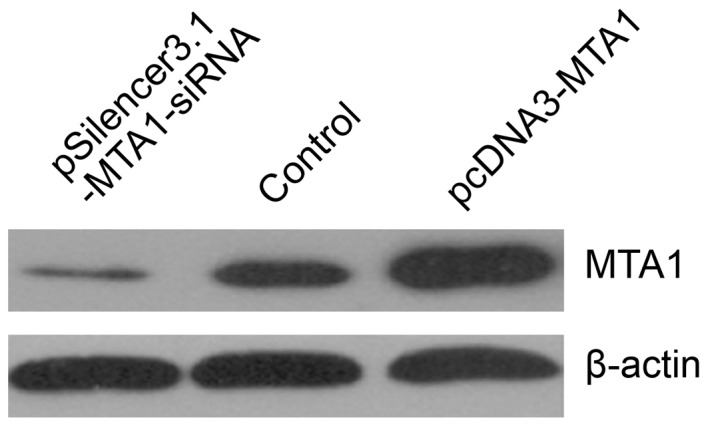
Western blot analysis of *MTA1* expression after transfection with pcDNA3-*MTA1* and pSilencer3.1-*MTA1*-siRNA. pcDNA3-*MTA1* and *MTA1*-siRNA were transfected into the cell line with Lipofectamine 2000. Whole protein was extracted and loaded for SDS-PAGE separation. The protein was transferred to a nitrocellulose membrane and pcDNA3-*MTA1* increased *MTA1* expression and *MTA1*-siRNA greatly decreased *MTA1* levels. *MTA1*, metastasis-associated gene 1.

We performed gene transfection and RNA interference to study *MTA1* functions. RT-PCR and western blot analysis confirmed the changes in *MTA1* levels after pcDNA3-*MTA1* and pSilencer3.1-*MTA1*-siRNA transfection. We found that the expression of *MTA1* was either suppressed or increased at the mRNA and protein level after treatment. The mRNA levels corresponding to pcDNA3-*MTA1*, control-siRNA and *MTA1*-siRNA, presented by density value, were 2.78±0.046, 1.04±0.119 and 0.32±0.046, respectively. The protein levels were 2.69±0.267, 1.07±0.112 and 0.36±0.069, respectively. Compared with the control-siRNA-transfected cells, *MTA1*-siRNA significantly decreased the expression of *MTA1* (P<0.05), while pcDNA3-*MTA1* significantly enhanced *MTA1* expression (P<0.05) (Figs. 2 and 3).

### In vitro migration and invasion ability of HEP-2 cells following transfection of pcDNA3-MTA1 and pSilencer3.1-MTA1-siRNA

**Figure 4:**
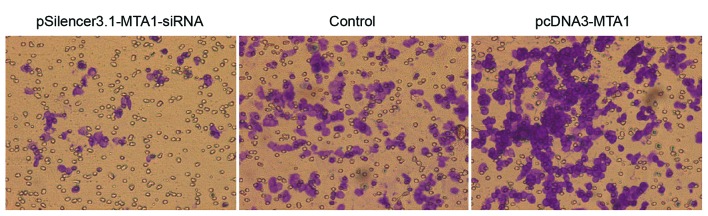
Migration ability of HEP-2 cells after *MTA1* overexpression and RNAi. Cells treated by *MTA1* overexpression and *MTA1* silencing were loaded into the upper wells of the chamber. Cells moved to the lower side of the fibronectin-precoated polycarbonate membrane were counted and analyzed. Compared with the control, pcDNA3-*MTA1* transfection promoted cell migration (P<0.05), while *MTA1*-siRNA transfection suppressed cell migration (P<0.05). *MTA1*, metastasis-associated gene 1.

**Figure 5:**
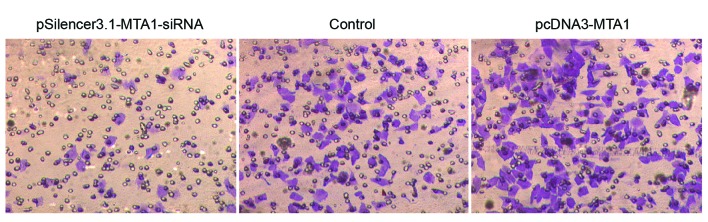
Invasion assay of HEP-2 cells after *MTA1* overexpression and RNAi. The model used in this experiment was the same as that used in the migration assay. The difference is that the membrane used in this experiment was coated with extracellular matrix Matrigel (250 μg/ml). Compared with the control, the number of cells that invaded through the membrane in the pcDNA3-*MTA1* transfection group was markedly higher, while that in the *MTA1*-siRNA transfection group was lower (P<0.05). *MTA1*, metastasis-associated gene 1.

The *in vitro* migration and invasion ability of HEP-2 cells transfected with pcDNA3-*MTA1* and *MTA1*-siRNA were studied using the Boyden chamber model as described in Materials and methods. In the migration assay, responding to pcDNA3-*MTA1*, control-siRNA and *MTA1*-siRNA, the number of cells that migrated to the lower side of the membrane was 549.2±21.51, 352±25.03 and 120.8±17.28, respectively. The number of cells that migrated to the lower side of the membrane in the invasion assay was 423.6±14.15, 301.2±25.4 and 115.4±15.52, respectively. Compared with the control-siRNA-transfected cells, *MTA1*-siRNA significantly decreased the migration and invasion ability of HEP-2 cells (P<0.05), while pcDNA3-*MTA1* significantly enhanced the migration and invasion ability of HEP-2 cells (P<0.05) (Figs. 4 and 5).

### Adhesion assay of pcDNA3-MTA1- and MTA1-siRNA-treated HEP-2 cell line

**Figure 6:**
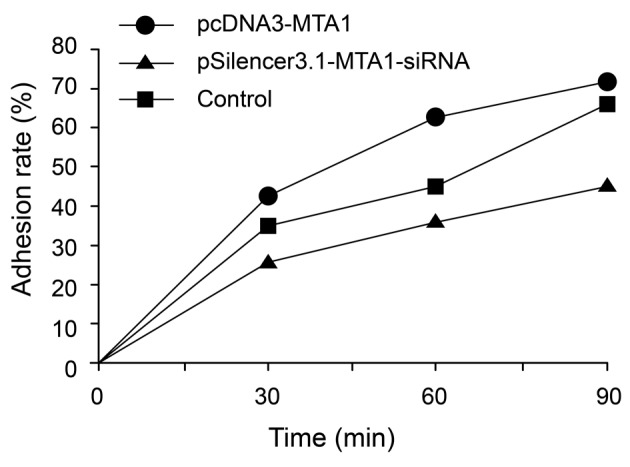
Adhesion assay of HEP-2 cells after *MTA1* expression and RNAi. Variously treated cells were seeded into Matrigel-coated 96-well plates. The plates were washed evenly for the indicated times. The attached cells were determined to reflect their adhesion ability. Compared with the control, pcDNA3-*MTA1* transfection promoted the adhesion of cells, while *MTA1*-siRNA transfection suppressed the adhesion of cells (both P<0.05, compared with the control). *MTA1*, metastasis-associated gene 1.

The adhesion of cancer cells to the extracellular matrix and cell surface molecules is a key step during metastasis *in vivo*. We evaluated the effects of the *MTA1* gene on the adhesion ability of cancer cells. The results showed that, at the early stage of adhesion, pcDNA3-*MTA1* promoted the adhesion of HEP-2 cells to the Matrigel matrix, while silencing of *MTA1* by RNAi inhibited the adhesion process. The adhesion rates of HEP-2 cells transfected with pcDNA3-*MTA1*, control-siRNA and *MTA1*-siRNA at 30 min post-cell seeding were 41.8±7.16, 35.6±6.08 and 26.6±2.97% (P<0.05, *MTA1*-siRNA vs. control and pcDNA3-*MTA1* groups), respectively; 63±10.44, 46±8.34 and 38.6±7.86% (P<0.05, *MTA1*-siRNA vs. control and pcDNA3-*MTA1* groups) at 60 min post-cell seeding, respectively; and 71.2±6.83, 63.6±7.56 and 45±6.08% (P<0.05, *MTA1*-siRNA vs. control and pcDNA3-*MTA1* groups) at 90 min post-cell seeding, respectively (Fig. 6). These results indicate that *MTA1* may exert its effects on metastasis by regulating the adhesion molecules on the cell surface.

### Wound healing assay

**Figure 7:**
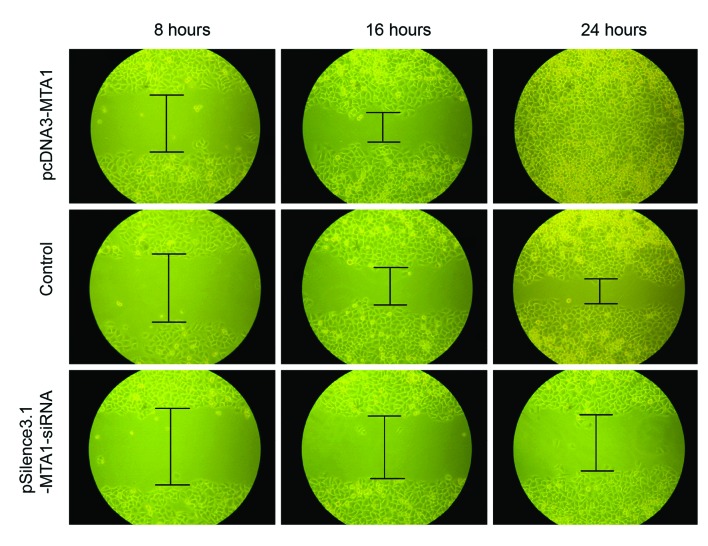
*MTA1* overexpression and RNAi changed the wound healing ability of the HEP-2 cell line. The variously treated cells were seeded into 35 mm dishes at the same density. The wound line was drawn in the cell layer with a sterile tip. The wound healing of cells differs corresponding to pcDNA3-*MTA1* or *MTA1*-siRNA transfection. At the designated time points, the healing of *MTA1*-siRNA transfected cells was significantly poorer. *MTA1*, metastasis-associated gene 1.

For the wound healing assay, equal numbers of transfected HEP-2 cells were reseeded into 35-mm-diameter culture wells. The wound healing ability of cells reflects their movement on the surface to which they are anchored for growth. At 8, 16 and 24 h after wounding, the healing ability of *MTA1*-siRNA-transfected cells was significantly poorer than that of the pcDNA3-*MTA1-* and control-siRNA-transfected cells (Fig. 7).

## Discussion

Cancer metastasis is the most common factor that causes cancer patient mortality and is a complex process involving a wide range of biological behaviors. It is urgent that the mechanisms underlying cancer metastasis be explored. Carcinogenesis involves several metastasis-associated molecules, many of which are altered during the process. Recent studies have revealed that *MTA1* is important in regulating cancer behaviors. *MTA1* expression has been associated with cancer malignancy in various cancer types, and has been found to increase the metastatic and invasive potential of carcinoma cells (12,31–33). LSCC is a type of cancer with a high potential for metastasis and invasion. To investigate whether *MTA1* is responsible, at least partially, for the metastatic potential of LSCC, *MTA1* gene function in the LSCC HEP-2 cell line was biologically studied using gene expression and RNAi techniques.

To confirm the correlation between *MTA1* and cancerous potential in LSCC cells, the *MTA1* level in HEP-2 and HaCaT cells was detected. It was observed that *MTA1* was expressed at relatively low levels in normal human keratinocyte HaCaT cell lines, while its expression was upregulated in HEP-2 cell lines. This result provided evidence supporting the function of *MTA1* in the development of human LSCC.

For further confirmation, gene transfection and RNA interference were performed to study *MTA1* functions in LSCC. RT-PCR and western blot analysis showed that the expression of *MTA1* was either suppressed or increased at the mRNA and protein level after pcDNA3-*MTA1* and pSilencer3.1-*MTA1*-siRNA transfection. To confirm the association between *MTA1* expression and the metastatic potential of cancer cells, migration, invasion, adhesion and wound healing assays were performed after manipulating *MTA1* expression. The results showed that overexpression of *MTA1* promoted the metastasis potential of HEP-2 cells, while *MTA1* silencing by RNAi reversed the malignant phenotypes. The *in vivo* metastasis ability of HEP-2 was inhibited by *MTA1* silencing. In the cellular biological studies, a causal correlation between *MTA1* expression and the *in vitro* migration and invasion ability of LSCC cancer cells was identified.

Silencing of *MTA1* also impairs the angiogenesis of prostate cancer, partially eliminating the circumstance of cancer growth (34). These studies support the idea that *MTA1* may be a potential target for cancer therapy. In the current study, silencing of *MTA1* reversed the cancerous behaviors of LSCC, extending the therapeutic value of *MTA1* in cancer treatment. *MTA1*-silencing was observed to result in a prominent loss of function in cancer cells, while overexpression of *MTA1* only increased cancerous behaviors to a certain extent. This may indicate that *MTA1* is critical in the life activity of cancer cells.

Molecular pathways were not the focus of the current study. Therefore, our next aim is to identify the mechanisms of *MTA1* responsible for the change in biological phenotypes and in the structure-function relationship of *MTA1*.

In brief, the results of the present study demonstrate that the expression of *MTA1* promotes the migration and invasion ability of HEP-2 cells, indicating its importance in the progression of LSCC. Suppressing HDAC activity is currently the target of chemotherapy (35–37). *MTA1*, as an HDAC component of NURD complexes, may be a potential powerful target for LSCC biotherapy or chemotherapy.
